# Global Cardiovascular Risk Assessment in the Management of Primary Hypertension: The Role of the Kidney

**DOI:** 10.1155/2013/542646

**Published:** 2013-07-29

**Authors:** Francesca Viazzi, Giovanna Leoncini, Roberto Pontremoli

**Affiliations:** Università Degli Studi e I.R.C.C.S. Azienda Ospedaliera Universitaria San Martino-IST, Istituto Nazionale per la Ricerca sul Cancro, 16125 Genoa, Italy

## Abstract

The knowledge of each patient's global risk profile is a prerequisite for effective therapeutic strategies in primary hypertension. Detecting the presence of subclinical organ damage at the cardiac, vascular, and renal levels is key for stratifying cardiovascular risk and may also be helpful in choosing antihypertensive agents and in monitoring the effectiveness of treatment. A systematic, in-depth search for subclinical organ damage, however, may be difficult to carry out because of logistic and economic problems related to the high prevalence of hypertension in the population. Renal abnormalities such as microalbuminuria and reduction in glomerular filtration rate have proven to be powerful predictors of cardiovascular and renal outcome. Thanks to their relatively low cost and wide applicability, more widespread use of these tests in the diagnostic workup will help detect subsets of patients at greater risk for whom additional preventive and therapeutic treatment is advisable.

## 1. Introduction

The prevalence and incidence of hypertension, arguably the most important modifiable risk factor for cardiac and cerebrovascular diseases, are going to increase dramatically worldwide over the next decade [1]. Prevention and treatment of high blood pressure (BP) already represent a public health challenge in many areas of the world and will likely require even more economic resources in the future. Not all hypertensive patients share the same adverse outcome, however. While, on the average, increased BP values are known to entail an unfavourable outcome whose magnitude is proportional to the severity of hypertension, for the majority of patients the long term risk of developing a cardiovascular (CV) event depends more on their overall risk profile than on their BP levels per se [2]. Given the overwhelming number of hypertensive subjects, early identification of those at greater risk for CV complications is of paramount importance because it could set the stage for directing additional measures to those who need them the most. Thus, besides taking into account traditional risk factors like age, gender, family history, obesity, smoking habits, lipid status, and diabetes, other conditions such as the presence of subclinical organ damage are currently used to identify high-risk patients and tailor treatment [3, 4].

## 2. Prognostic and Therapeutic Implications of Target Organ Damage

Subclinical organ damage at the cardiac, vascular, and renal levels often precedes and predicts the development of morbid events [5]. Thus, patients with left ventricular hypertrophy, especially the concentric type, show a higher risk of developing a coronary event or a stroke as compared to those with normal left ventricular geometry [6]. Similarly, carotid atherosclerosis has been associated with a worse prognosis regardless of other traditional risk factors. Noninvasive, ultrasound-detected left ventricular hypertrophy and/or asymptomatic signs of extracardiac atherosclerosis (i.e., intima media thickness at the carotid and femoral levels) are often used to identify subsets of patients at increased risk [7]. It has been shown that a systematic in-depth search for multiple risk factors or organ damage significantly increases the likelihood of identifying high-risk individuals [8]. However, given the large number of patients to be checked, logistic and financial reasons make this approach difficult, and routine application of these procedures is not currently recommended by international guidelines. On the other hand, an overly restrictive diagnostic approach to risk stratification could lead to significant misclassification of patients and to underestimation of the actual absolute risk, resulting in unfavourable practical and financial consequences (Table 1) [9].

Even more importantly, under effective antihypertensive treatment, changes in subclinical organ damage over time are paralleled by the modification of risk status [10, 11]. Thus, by noninvasively detecting the presence of left ventricular hypertrophy and/or carotid atherosclerosis, not only can we gather important information to help individualize treatment, but we can also monitor the effectiveness of treatment. It has also been shown that specific classes of drugs may exert additional organ protection beyond their BP-lowering effects [12–16]. A recent meta-analysis demonstrated that some classes of antihypertensive agents, such as those acting on the renin angiotensin aldosterone system or, in specific clinical settings, calcium antagonists, may provide regression of organ damage above and beyond what is expected by their BP-lowering effect, possibly through their specific mechanism of action [17].

## 3. The Kidney Message on Cardiovascular Risk

During the past several years, abnormal but minimal urinary albumin excretion, well below the threshold that is commonly detected by standard urinalysis (so-called microalbuminuria), has been shown to be associated with an unfavourable metabolic risk profile and with extrarenal signs of target organ damage, such as left ventricular hypertrophy and carotid atherosclerosis in patients with primary hypertension [18, 19]. A large body of data indicate that microalbuminuria is a strong, independent predictor of CV events both in patients with and without diabetes [20, 21]. While the exact pathophysiological mechanisms underlying the development of microalbuminuria are likely to be multifactorial and still not completely understood, it is generally agreed upon that this abnormality signals the coexistence of functional and structural abnormalities of the systemic vasculature secondary to atherosclerosis and hypertension. The resulting state of widespread increased permeability, which is revealed at the kidney level by an abnormal amount of urine albumin, possibly the end product of both increased glomerular permeability and reduced tubular reabsorption, is a forerunner and a risk factor for major CV events. A recent meta-analysis clearly showed that the risk for CV morbidity and mortality is linearly related to urinary albumin excretion and that the relationship becomes significant at relatively low values of albuminuria and shows no recognizable threshold or plateau [9, 22]. Furthermore, a reduction of albuminuria under antihypertensive treatment is paralleled by changes in CV risk [23, 24]. These results have led some investigators to claim that reducing albuminuria might become a therapeutic goal in itself.

Another subclinical renal abnormality, that is, a slight reduction in estimated glomerular filtration rate (eGFR), is also known to be an independent, powerful predictor of CV and global outcome [25]. This is an often overlooked but relatively common finding among hypertensive patients, at least in subgroups at greater risk for complications such as the elderly and patients with diabetes. As a matter of fact, several subclinical, asymptomatic but prognostically relevant changes may take place when eGFR drops to below 60 mL/min. Besides an increase in extracellular fluid volume and subsequent worsening of the haemodynamic load, other unconventional CV risk factors, such as anaemia, secondary hyperparathyroidism, vitamin D deficit, insulin resistance, endothelial dysfunction, hyperhomocysteinemia, subclinical inflammation, increased oxidative stress, and metabolic abnormalities in lipids and uric acid, have been described in the initial stages of renal disease and may in turn contribute to accelerating the progression toward atherosclerotic vascular damage and major events. 

**Figure 1:**
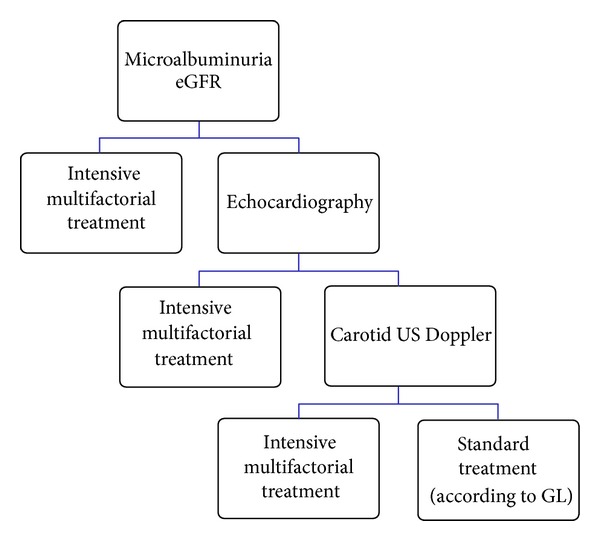
Looking for hypertensive target organ damage in clinical practice: the role of the kidney. Proposed diagnostic and therapeutic algorithm for the management of hypertension. A rational, cost-effective search for organ damage must start from low-cost, easy-to-perform tests and proceed to more expensive ones only in patients resulting at relatively low overall risk on the basis of previous risk stratification (modified from [27]).

Given the independent predictive power of the two renal markers, knowing one's level of urine albumin excretion provides additional prognostic information for almost any given value of eGFR and vice versa [9, 26]. A routine search for microalbuminuria and eGFR reduction may lead to the detection of a significantly higher percentage of patients with organ damage and yield a stratification of risk that is almost superimposable to what is obtained by the routine use of US, although at a significantly lower cost. This, in turn, may lead to a substantial improvement in identifying high-risk patients while optimizing the cost effectiveness of CV risk stratification [27]. Screening for the presence of these abnormalities is a relatively low-cost and therefore widely applicable way to implement a more thorough risk assessment of the hypertensive patient and gain useful information for therapeutic management. A rational, cost-effective search for organ damage must start from low-cost, easy-to-perform tests and proceed to more expensive ones only in patients resulting at relatively low global risk on the basis of previous risk stratification (Figure 1). In the presence of renal dysfunction or proteinuria, lower BP goals (i.e., 130/80 mmHg) are currently recommended [28].

Unfortunately, the powerful predictive power of renal abnormalities is not yet fully exploited in clinical practice, at least in Europe, as confirmed by a recent survey carried out by the ESH [29].

## 4. Conclusions

Thorough assessment of CV risk, including the presence and degree of target organ damage, is a prerequisite for devising effective therapeutic strategies and for tailoring treatment goals in primary hypertension. Clinical studies have shown that the higher the risk status of an individual patient, the greater the benefit for a given amount of BP reduction. The presence of target organ damage may also be helpful when choosing antihypertensive agents and in monitoring the effectiveness of treatment. Due to the high prevalence of high BP in the general population, logistic and economic reasons may limit a liberal approach to the evaluation of organ damage aimed at risk assessment. Subclinical renal abnormalities such as microalbuminuria or a slight reduction in eGFR provide a useful and easily applicable way to detect subsets of patients at greater risk for whom additional preventive and therapeutic treatment is advisable. A more widespread use of these tests in the assessment of CV risk in patients with hypertension is advisable.

## Figures and Tables

**Table 1:** ESH-ESC Guidelines 2013.

Marker	Predictive power (CV disease)	Feasibility	Cost effectiveness
Electrocardiography	+++	++++	++++
Echocardiography	++++	+++	+++
Carotid intima-media thickness	+++	+++	+++
Arterial compliance (pulse wave velocity)	+++	++	+++
Ankle-brachial index	+++	+++	+++
Coronary calcium score	++	+	+
Endothelial dysfunction	++	+	+
Cerebral lacunae/white matter disease	++	++	+
Estimated GFR	+++	++++	++++
Microalbuminuria	+++	++++	++++

The table shows how renal abnormalities, that is, increased albuminuria and reduced eGFR, are best suited for the initial routine assessment of cardiovascular profile in patients with primary hypertension (modified from [2]).

## Acronyms

BP: Blood pressure
CV: Cardiovascular
eGFR: Estimated glomerular filtration rate.
